# Frontal Cortical Asymmetry May Partially Mediate the Influence of Social Power on Anger Expression

**DOI:** 10.3389/fpsyg.2016.00073

**Published:** 2016-02-02

**Authors:** Dongdong Li, Changming Wang, Qin Yin, Mengchai Mao, Chaozhe Zhu, Yuxia Huang

**Affiliations:** ^1^State Key Laboratory of Cognitive Neuroscience and Learning, IDG/McGovern Institute for Brain Research, Beijing Normal UniversityBeijing, China; ^2^Center for Collaboration and Innovation in Brain and Learning Sciences, Beijing Normal UniversityBeijing, China; ^3^Beijing Key Laboratory of Mental Disorders, Beijing Anding Hospital, Capital Medical UniversityBeijing, China; ^4^Beijing Institute for Brain DisordersBeijing, China

**Keywords:** power, emotion, expressivity, frontal alpha asymmetry, mediator

## Abstract

When irritated by other people, powerful people usually tend to express their anger explicitly and directly, whereas people in less powerful positions are more likely not to show their feelings freely. The neural mechanism behind power and its influence on expression tendency has been scarcely explored. This study recorded frontal EEG activity at rest and frontal EEG activation while participants were engaged in a writing task describing an anger-eliciting event, in which they were irritated by people with higher or lower social power. Participants’ anger levels and expression inclination levels were self-reported on nine-point visual analog Likert scales, and also rated by independent raters based on the essays they had written. The results showed that high social power was indeed associated with greater anger expression tendency and greater left frontal activation than low social power. This is in line with the approach-inhibition theory of power. The mid-frontal asymmetric activation served as a partial mediator between social power and expression inclination. This effect may relate to the functions of the prefrontal cortex, which is in charge of information integration and evaluation and the control of motivation direction, as reported by previous studies.

## Introduction

As social creatures, human beings need to adapt their behaviors to certain social rules. One of the basic forces that shape social behaviors is power. This fundamental concept in social science (Russell, 2004) can be defined as an individual’s relative capacity to modify others’ states by providing or withholding resources or administering punishments (Keltner et al., 2003). The resources and punishments can be both material (food, money, economic opportunity, or physical harm) and social (knowledge, affection, decision-making opportunities, or ostracism). The higher capacity the individual has to deliver resources or punishments to others, the greater power he/she has. As a basic force in social life, power affects people’s social behavior greatly, including emotion expression behavior which is of interest by the current study. There have been many behavioral studies about the influence of social power on emotional expression/suppression (e.g., Garcia et al., 2007; Diefendorff et al., 2010; Langner et al., 2012), but surprisingly, little is known about the neural basis underlying the impact of social power on emotional expression/suppression. Previous studies (e.g., Keltner et al., 2003) have indicated that people with more power are more likely to express their emotions directly if they like so, which is associated with the function of behavioral approach or activation system. Some studies (e.g., Boksem et al., 2012) have suggested that social power may influence approach-related neural activities, such as those represented by frontal alpha asymmetry (FAA). As addressed by Davidson (1993), frontal EEG asymmetry may reflect the brain activities that moderate or mediate approach and withdrawal tendencies in responding to stimuli. In addition, the link between frontal asymmetry and emotion expression tendency has also been indicated by some studies (e.g., Harmon-Jones et al., 2003). Based on previous studies, we suppose that the neural mechanisms represented by FAA may play an important role in the influence of power on social behaviors. Thus the current study aimed to explore the approach/inhibition-related neural mechanism underlying the social power impact on emotion expression, with FAA as index. The research background of the relationships between social power, emotion expression, and frontal asymmetry is introduced below.

### Influence of Social Power on Social Behaviors

As implied by the concept of social power (Keltner et al., 2003), elevated power is associated with increased rewards and freedom and thereby activates approach-related tendencies (such as being more vigilant to rewards and being more aggressive). While reduced power is associated with increased threat, punishment, and social constraint and thereby activates inhibition-related tendencies (such as being more vigilant to threats, inhibition of opinion expression and emotion display). This perspective gets support from a large body of literatures. For example, a study (Kanso et al., 2014) found that power-holders, compared with subordinates, had greater expectation of rewards but reduced subjective magnitude attributed to losses. High-power individuals tended to take more risks which was mediated by optimistic risk perceptions (Anderson and Galinsky, 2006). Individuals of high power took more revenge on offenders, which was modulated by the justice concern (Kim et al., 1998) and individual differences (Strelan et al., 2014).

Besides the attitude to rewards/punishments and aggressive behaviors, social power also influences opinion and emotion expression that is of interest by the current study. For example, one study (Berdahl and Martorana, 2006) manipulated social power in three-person discussion groups and found that high-power individuals were more likely than low-power individuals to openly express their opinions. Social power affects not only opinion expression but also the expression of emotions. A study (Diefendorff et al., 2010) on display rules found that people exhibited more control over their emotional expressions when their interaction partner was higher in power compared with when their partner had equal or lower relative power. Additionally, studies (Garcia et al., 2007; Langner et al., 2012) about social class came to similar conclusions: people from lower-class backgrounds were less expressive and suppressed their emotions more. In contrast to the large body of behavioral studies about the influence of social power on emotional expression/suppression, the neural mechanisms of the influence of power on emotion expression are still poorly understood at present.

### The Neural Correlates of Social Power

There have been some reports, although not many, on the neural correlates of social power, which show that individuals in different power levels may have different neural activity patterns, specifically, the powerful persons manifest more approach-related neural activities. It is reported that when participants processed the faces of players in different social rank, the amplitudes of late positive potential differed among different ranks, and high-rank faces were associated with the highest reduction of alpha power (Breton et al., 2014). The left prefrontal cortex may be involved in the processing of power-related social motivations (Quirin et al., 2013). When participants were primed to a high or low social power situation, it was found that higher social power was associated with greater left frontal brain activation compared with lower social power (Boksem et al., 2012). When participants lower in social status processed social information, they were more likely engage neural structures (such as the dorsal lateral prefrontal cortex) often involved in mentalizing function (Muscatell et al., 2012) and goal-directed bebavior (Boksem et al., 2012).

### The Neural Correlates of Emotion Expression

Expressing emotion is regarded as a kind of approach behavior while suppression as inhibition or avoidance behavior, both related to different neural patterns. In an ERP study, participants with high anger-out scores and high anger-in scores showed divergent ERP patterns when they were conducting an emotion-word Stroop task (Stewart et al., 2010). Measures on infants found that relatively greater left frontal activation occurred during angry expressions, and the intensity of emotion expression was related to a generalized frontal lobe activation (Dawson, 1994). Besides the actual expression behavior, there have also been some reports of the neural correlates of emotional expression motivation. For examples, Hewig et al. (2004) found that subjects with higher left than right resting frontal cortical activity had higher anger expression scores and lower anger control scores, which support the hypothesis that emotion motivation direction (approach or withdrawal) is related to frontal asymmetry. Harmon-Jones et al.’s (2003, 2006) study revealed that left frontal activation was greater than right side activation when participants believed they could act on their anger as compared to when they believed they could do nothing, because their approach motivation in the action-possible condition was higher than that in the action-impossible condition. Similarly, Zinner et al. (2008) reported that, under special circumstances in which anger was not allowed to be expressed freely, anger was associated with withdrawal motivation and, hence, relative right-sided frontal activation. When manipulating frontal asymmetry directly with transcranial direct current stimulation, it was found that participants with increased left-sided frontal activation behaved more aggressively when they were insulted (Hortensius et al., 2012).

### Approach/Inhibition Motivation and Frontal Alpha Asymmetry

From literatures reviewed above, it can be seen that when researchers were addressing the influence of social power on emotion expression, and the neural correlates of power and emotion expression, they paid high attention to the approach and inhibition (also called withdrawal or avoidance) theory. This theory proposes that behavior of human and even non-human animals is driven by two fundamental motivational systems: approach and inhibition (e.g., Carver, 2001; Shah and Higgins, 2001; Zinner et al., 2008; Carver and Harmon-Jones, 2009; Rajchert and Winiewski, 2016). The approach system promotes behaviors such as purchasing rewards and opportunities, and making attacks. The inhibition system guides behaviors related to avoiding hurts, and being sensitive to punishments and social constraints. In the researches for neural correlates of approach/inhibition motivation or behavior, the behavioral approach system has been linked to relative left frontal cortical activity, and the behavioral inhibition system to relative right frontal cortical activity (e.g., Sutton and Davidson, 1997; Wacker et al., 2008; Harmon-Jones et al., 2010). Among this line of researches, frontal EEG asymmetry is a frequently used neural marker. Alpha power is most typically examined in the analysis of frontal EEG asymmetry, and is regarded as an index of the inverse of cortical activity. FAA is typically calculated by subtracting the natural log of left hemisphere alpha power from the natural log of right hemisphere alpha power (ln [right alpha] – ln [left alpha]). An increase in FAA reflects greater activation in the left side of the frontal cortex, and a decrease is related to greater right side activation. It is noteworthy that, by FAA, some literatures mean the state-related asymmetric activation elicited by stimuli or tasks (e.g., Harmon-Jones et al., 2003; Boksem et al., 2012), while some others mean the frontal asymmetries at rest (e.g., Hewig et al., 2004). The state-FAA possibly reflects the brain activation that is associated with, and even meditates, state response prompted by approach and withdrawal motivation. The resting-FAA is a trait-like EEG activity which is an individual difference variable related to, and even moderating, trait-tendency to approach and withdraw from stimulation (Davidson, 1993).

### Hypotheses

To explore the neural correlates of social power’s impact on emotion expressivity, the current study took anger expression as an example of emotion expression. Participants were asked to write about a scenario in which they were irritated by people who had more or less power than them, and to report their expression tendency. The studies reviewed previously indicated that social power exerts an influence on emotional expression, and frontal EEG asymmetry (as a measure of approach-inhibition motivation) may be linked to power manipulation and emotional expression, respectively. We therefore hypothesized that, when individuals in high social power situations are irritated by others in relatively less powerful situations, they would possibly have greater anger expression tendencies and more left sided frontal cortical activation. Furthermore, it is possible that the influence of social power is mediated by the mechanism represented by lateralized state frontal activation. In addition, the current study examined whether FAA at rest predicts anger expression tendency in anger evocative situations, and furthermore, whether resting-FAA moderates the effect of social power on anger expression.

## Materials and Methods

### Participants

Twenty-nine students (13 men and 16 women) participated in this experiment. Data from three participants were excluded from further processing and analysis, including one participant who blinked too much and two participants whose signals were too noisy to analyze. The remaining participants included 13 males (aged between 20 and 29, *M* = 23.62, *SD* = 2.60) and 13 females (aged between 20 and 27, *M* = 22.77, *SD* = 1.79). This study used a within-subject design, thus, all participants participated in both the high and the low power tasks (see “Preparation for social power priming” and “Procedure”). All participants received remuneration upon completion of the study. There have not been seen studies about social power impact on anger expression mediated or moderated by FAA. Due to the lack of effect size for reference, it is hard to estimate the appropriate sample size for the current study. But it is worth noting that a bigger sample size than the current used will certainly benefit the statistical power (see also “Discussion”).

Each participant was right-handed, which was verified with the Handedness Questionnaire (Li, 1983), and had normal or corrected-to-normal vision. They reported no history of neurological or mental problems. Written informed consent was obtained from each participant before the start of the experiment. This study was approved by the research ethic committee of the School of Brain and Cognitive Sciences in Beijing Normal University. All study procedures were in line with the latest version of the Declaration of Helsinki.

### Preparation for Social Power Priming

To induce participants into an anger-eliciting situation and manipulate the social power level, a writing task adapted from Boksem et al. (2012) was used, serving to prime high or low power. To enhance the sense of reality and gain a successful experimental manipulation, 1 day before the formal experiment, each participant was asked to describe some situations involving anger that had happened in their life. It aimed to find two events with similar scenarios (e.g., misled by a teacher vs. by a lower-grade student) but involving different interacting persons with higher or lower social power than the participants themselves. The experimenter judged if the events were suitable for use in the current experiment considering these aspects: whether there was a sharp contrast of social power between two events; whether they were very anger-evoking things according to the participant’s description and the experimenter’s life experiences; whether the two events had roughly similar plots except for the power comparison. Then the experimenter and the participant would try to reach a consensus on what events to be used in the formal experiment. During the formal experiment, the participants would be asked to write down the pair of events decided in the preparation stage (see “Procedure”). Participants who failed to provide appropriate anger-inducing events would not enter the formal experiment. We checked the written materials obtained in the study to see if the participants were compliant with the design intent of the writing task. We also counted the total words (TW) in each essay and calculated words per sentence (WPS) as an estimation of language use and hand movement during the writing task.

### Emotional State Measures by Self-Reports and Independent Raters

#### Initial Emotional State

A nine-point visual analog Likert scale (1 = very positive, 9 = very negative) was used to measure each participant’s emotional state before the writing task.

#### Anger Experience

Immediately following the writing task, the participants were asked “how angry did you feel in that situation?” A nine-point visual analog Likert scale (1 = not at all, 9 = a great deal) was used to measure each participant’s anger level. Two independent raters (one female, 21 years old; one male, 22 years old), who had no idea of the current research purpose, were also recruited to identify how angry the author was based on the written materials on the same Likert scale. The rater-reported anger levels were averaged across two raters as the final raters’ score for further statistical analyses.

#### Anger Expression Tendency

After the self-report of the anger experience, the participants were asked “will you express your anger explicitly in that situation?” A nine-point visual analog Likert scale (1 = not at all, 9 = a great deal) was used to measure each participant’s inclination toward expressing anger. Two independent raters rated the participants’ anger expression tendency with the written essays on the same nine-point Likert scale. The raters’ averaged sores were then passed to subsequent statistical analyses.

### Procedure

The participants were first instructed to read brief instructions describing the experimental process, and then the experimenter explained the procedure in detail. The experimenter would again confirm the events to be written in the experiment with the participants. Upon reaching an agreement on the situations to be written, the formal experiment began.

The participants were seated in a quiet chamber and received experimental prompts and instructions from a computer monitor in front of them. The formal experiment included six steps. *The first step* was resting. The participants were asked to close their eyes and rest for 2 min, and then open their eyes and fix on the “+” in the center of the screen for another 2 min. After resting, the participants reported their current emotional state on Likert scales. Immediately after self-reporting, the participants started *the second step*, in which they were asked to write about the anger-inducing event with the interacting person of higher or lower social power than themselves that was selected before the experiment.

In the high power condition, the instructions were as follows: *please imagine a specific event. In this event, a person with a lower social status than you made you very angry. The person has fewer resources, privilege, authority, or influence. He/she can be your subordinate, a group member of your team, and a lower grade student, etc.*

In the low power condition, the instructions given were as follows: *please imagine a specific event. In this event, a person with a higher social status than you made you very angry. The person has more resources, privilege, authority, or influence. He/she can be your boss, teacher, and team leader, etc.*

The participants had 8 min to imagine and write down the whole event, and they could press the enter key to continue if they finished ahead of time. The participants were ensured that all written materials would be used for scientific research only and that the contents shared in the study and identity information would not be leaked to the public at any time. In the writing, they described their emotional experience, language and behavior in the event as concretely as possible. They were then asked to report their anger and expression tendency scores. *The third step* was to write down the reason for their expression inclination. They had 3 min to write down why they were inclined to express or suppress their anger. Again, if they finished writing in advance, they could press the enter key to continue. After finishing these steps, the participants could take a short break to calm their emotions. When they felt they had returned to a calm state after the previous anger-eliciting event, they could press the enter key to continue. *The fourth step* was the same as the first step. The participants were asked to rest both with their eyes open and with their eyes closed. They then reported their current emotional state. In *the fifth step*, as in the second step, the participants were asked to write down a similar anger-eliciting event in which the interacting person had a different social power level. After the 8-min writing period, they reported their anger and expression inclination scores. In *the last step*, as in the third step, the participants were asked to write about how they considered whether to express their emotion.

The sequences of high and low power tasks were balanced across the 26 valid participants. Half of the participants imagined that they had more social power than their opponent in the first writing task and then less social power in the second writing task. The other half wrote about the event in which they had lower social power first and then more power second. While the participants were working on this task, their EEG signals were recorded simultaneously.

### EEG Acquisition and Reduction

The EEG data were recorded with a 128-channel Geodesic Sensor Net, the Electrical Geodesic Instrument (EGI) system (Tucker, 1993). The electrodes were placed in an extended 10–20 international system and referenced to Cz during recording. Oﬄine, all EEG activity was re-referenced to a global average reference. Horizontal electro-occulogram (EOG) was recorded with two electrodes placed at the outer canthi of both eyes. Vertical EOG was recorded with electrodes on the infra-orbital and supra-orbital regions of both eyes. All impedances were kept below 50 kΩ. The EEG from each electrode site was digitized at 500 Hz and was band-pass filtered between 0.01 and 200 Hz.

The raw EEG data were then down-sampled to 250 Hz and band-pass filtered between 0.5 and 35 Hz. Independent component analysis (ICA) was used to identify and correct the EOG artifacts. Then, the EEG file was visually inspected for movement artifacts, clipped signals and other sources of artifact. If artifact was present in any one channel at some time points, data from all channels were removed from further analysis at those time points. All of the above oﬄine analyses were performed using EEGLAB 9.0.4.4b (http://sccn.ucsd.edu/eeglab/), a Matlab-based open-source toolbox (Delorme and Makeig, 2004).

Eight pairs of homologous electrodes were selected for subsequent processing: FP1 and FP2 (frontal pole), F3 and F4 (mid-frontal), F7 and F8 (lateral-frontal), C3 and C4 (central), T3 and T4 (anterior-temporal), P3 and P4 (mid-parietal), T5 and T6 (posterior-temporal), and O1 and O2 (occipital). At each site of interest, a 1-min artifact-free EEG segment was selected for each resting stage (eyes-open and eyes-closed, respectively) and the event writing stage. EEG segments were subsequently divided into segments of 10 s each with 50% overlap. A continuous wavelet transformation (CWT) was used to estimate the spectral power density (μV^2^/Hz) in the alpha (8–12 Hz) band. The average alpha power density values for each stage at each site were then transformed using a natural log function. A measure of EEG hemispheric asymmetry was then derived (ln[right alpha]-ln[left alpha]) for each participant for each condition and each electrode pair. For the state-FAA, the participants’ alpha power density during the eyes-open resting stage was used as a baseline. We subtracted the baseline from the corresponding writing stage and used the difference scores as state-FAAs in subsequent statistics. Pearson correlation analyses indicated that the resting-FAA measures had high stability across various resting stages. At the mid-frontal area (represented by F4 and F3), the resting FAA for eyes-closed stage (*M* = -0.013, *SD* = 0.531) correlated positively with that for eyes-open stage (*M* = -0.031, *SD* = 0.555), *r* = 0.912, *P* < 0.001; the first-time resting FAA (*M* = -0.031, *SD* = 0.592) correlated significantly with the second-time (*M* = -0.013, *SD* = 0.487), *r* = 0.934, *P* < 0.001; the resting FAAs for high power (*M* = -0.069, *SD* = 0.508) and low power (*M* = 0.025, *SD* = 0.570) conditions correlated to each other as well, *r* = 0.937, *P* < 0.001. In subsequent statistics, the resting-FAA was an average across the eyes-open and the eyes-closed resting stages.

### Statistical Analyses

All statistical analyses were performed in SPSS v.20 (SPSS Inc.). First, we conducted a series of paired-samples *t*-test between the high and the low power conditions on these indexes: TWs, WPS, initial emotional state, anger level and expression tendency by self-reports and observer-ratings, and state frontal alpha asymmetries (on most typically used F4-F3 pair, with P4-P3 pair as contrast). One extreme state-FAA score in the distribution of EEG asymmetry was replaced by a value of mean + 2.5 *SD* before the analyses, as suggested by previous literatures (Rivest, 1994; Meyer et al., 2014). For estimating the inter-rater reliability of anger level and expression tendency, the intraclass correlation coefficient (*ICC*) was used for quantifying the consistency of the emotional state measures between self and observers (average score), and between the two observers. *ICCs* for single measure (*ICCs*) and average measure (*ICCa*) were calculated based on a two-way random effect model for consistency measurement (McGraw and Wong, 1996). The reliability levels quantified by *ICC* were graded according to the criteria proposed by Cicchetti and Sparrow (1981), wherein the reliability having a value of >0.75 is considered as “excellent,” 0.59–0.75 as “good,” 0.40–0.58 as “fair,” and <0.40 as “poor.” Second, we assessed the mediation effect due to state-FAAs that might vary between the social power conditions. To avoid the demand effects, the observer-rated expression tendency scores were used in the mediation analyses and the following moderation analyses. We used the method proposed by Judd et al. (2001), which is an analytic approach for examining mediation and moderation in within-subject designs. The anger expression score would be regressed on the state FAA under the high and low power conditions, respectively. If the association between FAA and expression inclination was confirmed, the expression inclination difference between the high and low power conditions would be regressed on the FAA sum and difference. If the FAA difference could be predictive of the expression difference, FAA could be said to mediate the impact of social power on emotion expression. Then the residual impact over and above mediation would be estimated. Finally, the moderation effect of resting-FAA was estimated with the methods suggested by Judd et al. (2001). The anger expression score would be regressed on the resting FAA under the high and low power conditions, respectively. Then the expression differece would be regressed on resting-FAA. If the slope for resting-FAA was significantly different from zero, it meant that there was a significant resting-FAA × power interaction, equivalently, resting-FAA moderated the power influence on expression tendency. Alpha was set at 0.05 for all analyses.

## Results

### Contents of Writing

Through inspection of the participants’ written materials, it could be seen that all the participants had complied with the beforehand agreement to imagine the anger-eliciting events that had been discussed and chosen with the experimenter. The essays written by same participant under the high power and the low power conditions had similar lengths (see **Table 1**). The social power was also manipulated appropriately. In those events the participants were irritated by persons either more powerful or less powerful than themselves. For example, an adviser or a lower-grade schoolmate was absent for an appointment without notice. Although the participant was similarly angry in both scenarios, the social power contrast might lead to different anger expressing inclinations. The results showed that when the participants were in a high power condition (e.g., the appointment was with a lower-grade schoolmate), most of them (23/26) were more inclined to express their anger directly. The reasons they reported were as follows: venting anger, teaching the person a lesson, feeling a sense of superiority and the privilege of a high position, having no negative consequences and promoting the development of projects. The first two reasons were mentioned most frequently. However, when the participants were in a low power condition (e.g., the appointment with his/her adviser), most of them (23/26) chose low expression behaviors for the following reasons: keeping the relationship in positive standing for the future, respecting elders, understanding and feeling sympathy for others’ difficulties, not being able to find the appropriate expression behaviors and not being able to change the outcome. The first two reasons were mentioned most frequently.

**Table 1:** Paired-samples *t*-tests of written essays’ total words (TW) and words per sentence (WPS) between the high and low power conditions (*n* = 26).

	High power (*M* ±*SD*)	Low power (*M* ±*SD*)	*t*-value	*P*-value
TW	214 ± 69	222 ± 80	-1.239	0.227
WPS	26 ± 4	25 ± 3	0.477	0.638

### Emotional State

The paired-samples *t*-test indicated that when comparing the participants’ initial emotional states before writing, no difference was found between the high power (*M* = 5.50, *SD* = 0.76) and the low power (*M* = 5.42, *SD* = 0.95) conditions, *t*(25) = 0.386, *P* = 0.703. On both the self-reports and the observer-ratings, the participants’ anger levels evoked by the events that they wrote about showed no significant difference between the high and the low power conditions (see **Table 2**). The inter-rater consistency had fair to excellent reliability under the high and low power conditions (see **Table 4**).

**Table 2:** Paired-samples *t*-tests of participants’ anger levels during the writing tasks between the high and low power conditions (*n* = 26).

	High power (*M* ± *SD*)	Low power (*M* ± *SD*)	*t*-value	*P*-value
Self-report	7.31 ± 0.84	7.58 ± 0.86	-1.272	0.215
Observer-rating	7.12 ± 1.03	7.27 ± 1.08	-0.527	0.603

### Expression Inclination

As shown in **Table 3**, on both the self-reports and the observer-ratings, the participants’ anger expression tendency were significantly higher in the high power condition than in the low power condition. And under the two conditions, *ICCs* also showed high inter-rater consistency of anger expression inclination (see **Table 4**).

**Table 3:** Comparisons of participants’ anger expression tendency between the high and low power conditions (*n* = 26).

	High power (*M* ± *SD*)	Low power (*M* ± *SD*)	*t*-value	*P*-value
Self-report	7.04 ± 1.54	3.00 ± 1.63	9.211	*P* < 0.001
Observer-rating	7.08 ± 1.52	3.42 ± 1.50	8.729	*P* < 0.001

**Table 4:** Inter-rater reliability of anger level and anger expression tendency estimated with *ICC* (*n* = 26).

	Anger level				Anger expression			
	High power		Low power		High power		Low power	
	*ICCs*	*ICCa*	*ICCs*	*ICCa*	*ICCs*	*ICCa*	*ICCs*	*ICCa*
Self and Raters	0.637 (<0.001)	0.778 (<0.001)	0.546 (0.002)	0.706 (0.002)	0.855 (<0.001)	0.922 (<0.001)	0.850 (<0.001)	0.919 (<0.001)
Rater 1 and Rater 2	0.460 (0.008)	0.631 (0.008)	0.438 (0.011)	0.609 (0.011)	0.489 (0.005)	0.657 (0.005)	0.545 (0.002)	0.705 (0.002)

*ICCs, single measure; *ICCa*, average measure. The exact *P*-values were in the parentheses.*

### State Frontal Alpha Asymmetry

**FIGURE 1:**
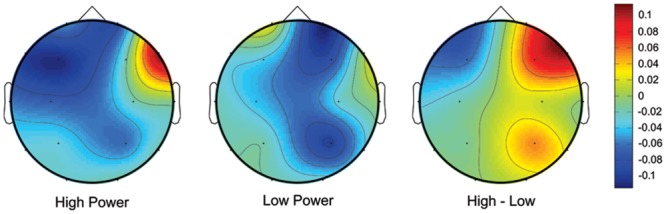
**Scalp topographical maps of state-related alpha power density (μV^2^/Hz) for the high and low power conditions.** A difference map by high minus low power condition was also shown.

At the mid-frontal area (represented by F4 and F3), the paired-samples *t*-test on FAAs induced by the power priming task showed that there was greater left-sided frontal activation in the high power condition than in the low power condition, while there was no significant difference of EEG asymmetry between the two conditions at the parietal area (see **Table 5**). **Figure 1** showed the alpha asymmetries in a whole scalp view.

**Table 5:** Comparisons of participants’ EEG asymmetries (μV^2^/Hz) at F4-F3 and P4-P3 electrode pairs between the high and low power conditions (*n* = 26).

	High power (*M* ±*SD*)	Low power (*M* ±*SD*)	*t*-value	*P*-value
F4-F3	0.091 ± 0.287	-0.063 ± 0.247	2.653	0.014
P4-P3	-0.029 ± 0.288	-0.093 ± 0.289	1.050	0.304

### Mediation Effect

**FIGURE 2:**
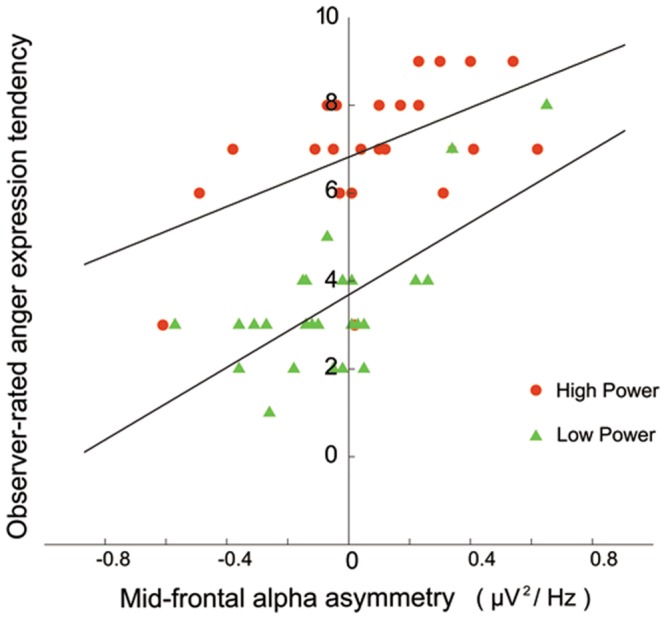
**Relationship between the observer-rated anger expression tendency and the state frontal alpha asymmetry (FAA) measured at F4-F3 electrode pair for the high and low power conditions, respectively**.

As described above, the total effect (estimated by the mean difference of expression tendency, which equaled 3.66) of social power on expression tendency was significant and the effect of social power on state-FAA (the potential mediator) was also significant. When the anger expression score was regressed on the state mid-frontal asymmetry for the high and low power conditions, the state-FAA in each condition was significantly related to expression scores, with higher left frontal activation associated with higher expression inclination (see also **Figure 2**): for high power, β = 0.532, *t*(24) = 3.075, *P* = 0.005, *R*^2^_adjusted_ = 0.253; for low power, β = 0.679, *t*(24) = 4.530, *P* < 0.001, *R*^2^_adjusted_ = 0.439. When the expression difference was regressed on state-FAA sum and difference, state-FAA difference was a significant predictor of expression difference (i.e., the state-FAA level might mediate the social power influence on expression tendency): β = 0.559, *t*(23) = 3.270, *P* = 0.003, *R*^2^_adjusted_ = 0.288, *r*^2^_partial_ = 0.317. In addition, after controlling for the mediation effect, the direct effect of social power on anger expression tendency (estimated by the mean difference of expression tendency after centering the sum of asymmetry, which equaled 3.03) was still significant, *t*(23) = 7.562, *P* < 0.001, which meant the state-FAA exerted as a partial mediator in the social power influence on anger expression tendency.

### Moderation Effect

When the anger expression score was regressed on resting-FAA for the high and low power conditions, frontal asymmetry was a marginal significant predictor of expression under the high power condition: β = -0.388, *t*(24) = -2.062, *P* = 0.050, *R*^2^_adjusted_ = 0.115, while under the low power condition, resting-FAA was not related to anger expression tendency: β = -0.141, *t*(24) = -0.700, *P* = 0.491, *R*^2^_adjusted_ = -0.021. When the expression difference was regressed on resting-FAA, the slope for asymmetry was not significantly different from zero: β = -0.177, *t*(24) = -0.881, *P* = 0.387, *R*^2^_adjusted_ = -0.009, meaning that the resting-FAA × power interaction was not significant, i.e., the resting-FAA might not moderate the expression difference between the high and low power condtions.

## Discussion

This study explored social power influence on anger expression inclination and the potential underlying neural basis. The participants’ expression score (by both self-reporting and observer-rating) and state frontal alpha asymmetries differentiated between the high and the low power conditions. The state-FAA partially mediated the influence of social power on expression inclination. The current study failed to find the resting-FAA as a predictor of anger expression.

### Motivation Directions may Underlie the Social Power Influence on Anger Expressivity and Frontal Activation

Participants in this study had similar levels of anger in the high and the low power conditions, but their anger expression inclinations were different between the two conditions. The high inter-rater consistency indicated by *ICCs* suggested that the current study had reliable measures of anger level and expression tendency. The self-reports and the observer-ratings had similar patters under various conditions. The written contents revealed that in the high power condition, the participants were more likely to express their anger because they wanted to vent the unpleasant emotion and teach the person who offended them a lesson. It has been evidenced that anger relates to an approach motivational system (for a review, see Carver and Harmon-Jones, 2009). This affect arises from a disruption of approach toward a desired goal, and it often promotes efforts to restore a desired state. Anger expression may help people to change the behavior of others and stop the violation of their desire. People with more power, as addressed by Keltner et al. (2003), are usually able to get access to more abundant resources and able to attain their important goals, and meanwhile, have less constraints of their actions. This situation may facilitate the activation of their approach systems greatly. Thus, it is not hard to understand the high expressing tendency of participants under the high power condition in the present study. However, in a social context, people do not always have the freedom to express their anger freely. In the low power condition of this study, most participants did not tend to show their anger explicitly. The main reason they reported was a fear that expressing their anger might bring about negative consequences because the interacting partners were more powerful than them. These results are consistent with some previous studies (e.g., Berdahl and Martorana, 2006; Diefendorff et al., 2010; Petkanopoulou et al., 2012) which also report lower expressivity in low power situations. Indeed, the lack of power will lead to a decrease in the probability of acquiring resources, and an increase of sensitivity to social constraints (Keltner et al., 2003). Expressing anger or teaching the offender a lesson may help to resolve the anger-eliciting event (Harmon-Jones, 2004), but a head-on confrontation may also induce negative consequences, especially for those in relatively lower social positions. According to the approach-inhibition theory of power (Keltner et al., 2003), the activation of inhibition motivation system may account for the low expressivity that is possibly more socially adaptable when people are in a lower power situation.

One then may ask how social power manipulates anger expression on a neural level. High power status not only led to higher expression inclination but also caused greater left-side frontal activation. This result is in agreement with the finding of Boksem et al. (2012). It should be noticed that the current study used a writing task (by right hand) to achieve power manipulation and anger elicitation. As reported by some studies (e.g., Harmon-Jones, 2006; Peterson and Harmon-Jones, 2008), the right-hand movement possibly caused greater left frontal cortical activation. However, as the results of analysis of written materials, the total length of essays and the average length of each sentence did not differentiate significantly between the high and the low power conditions, thus, the observed frontal activation difference between the two conditions is not likely to be attributed to a movement difference. According to Davidson et al.’s (1990) theory, the fundamental continuum along which frontal cortical regions are lateralized is approach-withdrawal, with the left frontal region serving as an approach system and the homologous right hemisphere region serving as a withdrawal system. Additionally, approach and withdrawal are components of different emotions. For example, anger will elicit the approach motivation and result in greater left frontal activation. In this study, the anger levels in the two power conditions were the same. Therefore, the frontal asymmetry difference is not likely due to a difference in anger levels between the two conditions. A more likely explanation is that the power level caused a manipulation of the behavioral response direction represented by asymmetric activation of the frontal cortex. From Keltner et al.’s (2003), power influences the balance between approach and withdrawal. High power activates approach-related processes, while low power activates withdrawal processes. In the current experimental context, participants of high power status would be less likely to restrain their words and deeds. In other words, they would express their opinions and attitudes more freely and more directly. This approach motivation is associated with greater left frontal activation. On the contrary, when participants were in a low power condition, they had to be sensitive to other people’s thoughts (e.g., Tullett et al., 2012, see also Muscatell et al., 2012) and act cautiously to not cause serious consequences. This resulted in greater activation of the withdrawal system.

### Social Power Effect May Be Based on Neural Mechanisms Represented by FAA

In addition to the associations between power and both expression and asymmetry, further analysis also showed evidence of the association between state-FAA and anger expression inclination. Meanwhile, a partial mediation effect of state-FAA was revealed by the current data. In other words, social power may influence emotional expression partially through the neural mechanism tapped by FAA. Although little has been known about the neural origins of FAA, the available evidence suggests that frontal cortical asymmetry may reflect activity in the prefrontal cortex, especially the dorsolateral prefrontal cortex (e.g., Boksem et al., 2012). In addition, it is proposed that the corpus callosum provides a possible neuroanatomical correlate for frontal cortical asymmetries and the interhemispheric signal transfer plays a role in the emergence of approach-related motivation and behavior (Schutter and Harmon-Jones, 2013). Previous studies have indicated that the prefrontal cortex is implicated in information evaluation (e.g., Ochsner et al., 2009; Sakaki et al., 2012; Hintze et al., 2014), decision making (e.g., Hutcherson et al., 2012; Weickert et al., 2014), and modulation of social behavior (e.g., Anderson et al., 1999; Perach-Barzilay et al., 2013). In social life, people may face complex social situations and sometimes have to manage conflicting information and desires. The prefrontal cortex plays an important role when people integrate and analyze information from the outer and inner environments, balance all parties involved in the game, and then finally choose appropriate social behaviors. In the current study, power influenced anger expression tendency greatly. The underlying neural mechanism might relate to the function of information analysis and behavior control (e.g., Barbas et al., 2011), which relies much on the flexible interaction within and between brain areas (e.g., Oehrn et al., 2014). It was revealed that phase synchrony of alpha oscillations could serve as a mechanism of brain communication (Billeke et al., 2014). The alpha asymmetry may also serve as the neural basis of interaction and coordination of left and right hemisphere when individuals try to adapt behaviors in socially demanding environments. When individuals were irritated by someone, the neural mechanism represented by FAA worked to integrate emotional experience with information including contextual memory and situational knowledge, to enact behavioral responses (Gatzke-Kopp et al., 2012). In these processes, immediate pleasure, long-term goals, the power contrast between two individuals, and social rules are considered as a whole, then people can choose preferred explicit behavior on the basis of these considerations.

Aside from the state fontal asymmetry, it has been suggested that the trait of asymmetry might moderate an individual’s characteristic emotional response, which is referred to as affective style by (Davidson, 1998a,b,c). Individuals may differ systematically and consistently when they respond to social events. However, the current study failed to find the resting-FAA to act as a moderator of the relation between social power and anger expression tendency, and also failed to find the association between resting frontal asymmetry and expression tendency. This result seems contrary to the reports (e.g., Harmon-Jones, 2004; Hewig et al., 2004; Huffmeijer et al., 2012) that indicate a link between frontal cortical asymmetry at rest and approach motivation/behavior. And there is indeed an inference that frontal EEG asymmetry may possibly function more robustly as a mediator of emotional responses than as a moderator (Coan and Allen, 2004). But, a denial of the FAA-approach link and the moderation effect of resting frontal asymmetry would be at great risk if just based on the current results. The large individual differences in the current study may influence the estimation of the FAA-expression association. In addition, although the resting-FAA had high stability in the short interval of current experiment, the asymmetry scores measured in only one occasion was not necessarily robust enough to represent a trait-like EEG characteristic. Some studies (e.g., Hewig et al., 2004; Kline and Allen, 2008) have suggested that, for more reliable estimates of trait-FAA, it would be better to aggregate recordings across multiple measures in several weeks apart. In addition, it is worth noting that, although the current study found a significant mediation effect of state-FAA on the social power impact on anger expression tendency, and the effect size (estimated with *r*^2^_partial_) is about medium with 0.25 representing a medium effect in the social science field (Ferguson, 2009), an adequate statistical power should be paid more attention to guarantee the repeatability of findings. Increasing the sample size is a preferred strategy for increasing statistical power (e.g., Fritz and MacKinnon, 2007). Furthermore, as mentioned above, the large individual differences in resting-FAA also indicates the necessity of a bigger sample size in the future studies interested in the mediation or moderation effect of frontal cortical asymmetry. And caution should be exercised regarding the current findings.

### Limitations and Future Directions

The current study is limited in several ways. Besides the above-mentioned issues about sample size and reliability of resting-FAA, the objectivity of the experiment also needs to be strengthened in future work. It is obvious that the experimental results might be influenced by the selection of anger-eliciting events and the self-reported anger levels and expression tendencies. In the current study, the experimenters who were in charge of communicating with the participants about events selection, and data acquisition, were not blind to the research purpose. Although they did not deliberately try to induce participants to behave toward the experimental prediction, a better way to avoid the subjective bias is to have a few executors, who are unaware of the experimental purpose, to conduct the interview for event selection and the data collection. For the emotional state measures, the current study limited the question to anger and its expression, which has a certain degree of probability that leads to biased self-reports. The observer rating used in the current study may help to reduce the bias. In addition, if participants get the opportunity to report their emotional intensity and expression tendency on multiple emotional options (such as happiness, anger, fear, disgust, sad, and surprise), the objectivity and comprehensiveness of emotion measurement would be improved. The current study did not measure explicit approach/inhibition behavior. As found in this study, participants were more likely to express their anger when facing a subordinate, but tended to express less to a superior. This finding, to some extent, is consistent with the phenomenon of displaced aggression (e.g., Vasquez, 2009; Scott et al., 2015; Rajchert and Winiewski, 2016). When a person is provoked but unwilling or unable to revenge on the original provocateur, he/she possibly behaves aggressively toward an innocent but assailable target, and the displaced aggressive behavior is related to the behavioral approach and inhibition system (Rajchert and Winiewski, 2016). The approach trait is positively correlated with displaced aggression, and the inhibition trait restrains direct aggression, but is a positive predictor of displaced aggression after controlling for the behavioral approach system. The task design of present study did not allow for an explicit observation of displaced and direct aggression behavior. Further work taking this issue into consideration would be of interest. Meanwhile, the identification of reasons for people to change their direct responses and corresponding consequences, such as emotion regulation strategies they may use and trade-off between short and long-term goals, would deepen the understanding of the role of social shaping. The current study took anger expression as an example of emotional expression. There are certainly other category of emotions, such as fear (having same valence but different motivational direction with anger, e.g., Wacker et al., 2003), determination (e.g., Harmon-Jones et al., 2011) and enthusiasm (having different valence but same motivational direction with anger, e.g., Harmon-Jones et al., 2010), and cognitive dissonance (same valence and same motivation, e.g., Harmon-Jones, 2004). Will the effect of social power vary with the emotion categories? Studies in a wider range of emotions would contribute to understanding of the role of power and the approach-inhibition theory. It is noteworthy that, studies based on the approach-inhibition model of power have primarily focused on whether power leads to more action or not. Approach behavior certainly involves action, but avoidance behavior, i.e., actively moving away from certain target, also involves action. Active avoidance is very different response from freezing-like inhibition, and may result in very different outcomes (Smith and Bargh, 2008). More attention of active avoidance is needed in future work interested in social power and the approach-inhibition theory. To understand the antecedents and consequences of environment-person interaction, much attention also need be paid to individual differences. Aside from the trait of frontal asymmetry, other factors such as personality are worthy of consideration. Specifically, in situations involving social power, a person’s dispositional power (e.g., Chen et al., 2009) may interact with situational power (e.g., Richeson and Ambady, 2003; Lonnqvist et al., 2011) and play a role in the final behavioral choice. Future work on personality processes and individual differences would enhance our understanding of the psychological and neural basis of social power. In addition, as mentioned before, little has been known about the neural origins of asymmetric activity of frontal cortex. More work is still needed to address the neural substrates of social power in the future.

In summary, the current study replicated the impact of social power on emotional expression and identified FAA as a possible mediator in that process. This effect may be dependent upon the frontal system, which plays a role in information integration and evaluation and behavior control. It is noteworthy that people’s social behaviors are governed by many factors. To achieve a comprehensive understanding of the interaction between social context and individuals, neural mechanisms, environmental conditions, individual differences and other related factors have to be considered systematically.

## Author Contributions

DL, YH, CW, and CZ designed the experiment. DL and MM performed the experiments. YH, CW, DL, and QY analyzed the data. All authors discussed the results and contributed to the writing of the paper.

## Conflict of Interest Statement

The authors declare that the research was conducted in the absence of any commercial or financial relationships that could be construed as a potential conflict of interest.
